# Exploring the Feasibility of an Insourced 3D-Imaging Reconstruction: Preliminary Series with the Use of Synapse3D™ Software

**DOI:** 10.3390/cancers18162692

**Published:** 2026-08-20

**Authors:** Daniele Fettucciari, Martina Bracco, Cristina Carerj, Filippo Gavi, Francesco Rossi, Giuseppe Pallotta, Simone Assumma, Enrico Panio, Alessandra Francocci, Vincenzo Cavarra, Marco Montesi, Nicoletta Testori, Pierluigi Russo, Filippo Maria Turri, Angelo Totaro, Carlo Gandi, Nazario Foschi, Lorenzo D’Amico, Francesco Pio Bizzarri, Emilio Sacco, Matteo Pavone, Giorgia Gaia, Bernardo Maria Cesare Rocco, Maria Chiara Sighinolfi

**Affiliations:** 1 Department of Urology, Fondazione Policlinico Gemelli Istituto di Ricovero e Cura a Carattere Scientifico-IRCCS, 00168 Rome, Italy; 2 Facoltà di Medicina e Chirurgia, Università Cattolica del Sacro Cuore, 00168 Rome, Italy; 3 Department of Urology, Ospedale Isola Tiberina-Gemelli Isola, 00168 Rome, Italy; 4 Department of Gynaecology, Fondazione Policlinico Gemelli IRCCS, 00168 Rome, Italy

**Keywords:** 3D reconstruction, insourcing, Synapse 3D, renal cell tumour, robotic surgery

## Abstract

**Background/Objectives:** To evaluate the feasibility and the learning curve of an insourced, clinician-performed three-dimensional (3D) image reconstruction workflow using Synapse3D™ software in a robotic urologic surgery setting. **Methods:** In this prospective single-centre case series, 20 consecutive patients scheduled for robotic kidney surgery were enrolled over a 30-day period. Three urology residents without prior experience in 3D reconstruction completed a structured two-day training programme including eight supervised practice reconstructions. Subsequently, each one reconstructed all 20 study cases independently. Reconstruction time was recorded for every reconstruction. After surgery, the two operating surgeons independently rated each model on a 5-point Likert scale for anatomical fidelity, defined as the correspondence between the model and the anatomy encountered intraoperatively. **Results:** All 60 reconstructions were completed successfully, with no failed reconstructions, no DICOM (Digital Imaging and Communications in Medicine) import errors and no software crashes; assistance from the manufacturer was never required. Mean reconstruction time was 19 min (range 11–29 min). Reconstruction time decreased by 0.63 min per case (95% CI 0.51–0.74; *p* < 0.001). Median time fell from 23 min in the first ten cases to 15 min in the last ten (*p* < 0.001), and the reduction was significant for each operator analysed separately. Anatomical fidelity was rated 4 (IQR 1) by both surgeons. The two surgeons rated overall system usability 3/5 and 5/5. **Conclusions:** Insourced 3D reconstruction with Synapse3D™ is technically feasible and is learned rapidly and reproducibly by urology residents after limited training.

## Simple Summary

Three-dimensional (3D) reconstruction of patient-specific renal anatomy is increasingly used to support planning in kidney surgery, but the models are usually produced by external providers. Synapse3D™ (V6.8.0017EU Build 0052, Fujifilm) is a clinician-oriented software package that allows a surgical team to generate 3D kidney models in-house from routine CT scans. In this study, three urology residents with no prior experience completed a two-day training programme and each independently reconstructed the same 20 consecutive cases scheduled for robotic kidney surgery. Every reconstruction was completed successfully. Reconstruction time fell by approximately 0.6 min for each additional case, and after approximately ten cases the mean time per case had fallen below 18 min; the rate of improvement was similar for all three residents. After surgery, the two operating surgeons reviewed each model and rated how closely it matched the anatomy they had encountered in the operative field. This study shows that in-house 3D reconstruction is technically feasible and can be learned quickly.

## 1. Introduction

Renal cell carcinoma (RCC) accounts for approximately 2–3% of all malignancies, with the highest rates of incidence observed in Western countries [1]. The rising incidence of renal cancer, largely attributable to advances in imaging and to incidental detection [2], has increased the demand for accurate and organ-preserving treatment strategies. Nephron-sparing surgery, in particular partial nephrectomy (PN), is a standard treatment for clinically localised T1 renal cell cancer, offering oncological control while preserving renal function [3,4]. The adoption of robot-assisted techniques has significantly expanded the use of PN to accommodate more complex tumours, enhancing surgical precision in dissection and reconstruction [5,6], and contemporary comparative series and meta-analyses have extended its indications to progressively more demanding scenarios [7,8]. This expansion is part of a wider technological transformation of genitourinary cancer surgery, in which artificial intelligence, robotics, 3D imaging and image-guided planning are converging [9]. Minimally invasive surgery has become increasingly prevalent in the management of RCC, driven by the aim of improving patient outcomes, reducing recovery times and minimizing postoperative complications. However, the complexity of kidney tumour anatomy—especially for endophytic or hilar tumours—can pose significant challenges for surgical planning and intraoperative orientation [10]. Traditionally, surgeons rely on two-dimensional (2D) cross-sectional imaging (CT or MRI) and mentally reconstruct three-dimensional (3D) relationships between tumours, vessels, and the collecting system. This mental reconstruction is heavily dependent on the surgeon’s spatial abilities, which can vary considerably among individuals, and can potentially lead to inaccuracies.

In response to these challenges, 3D virtual model reconstruction has emerged as a valuable tool to enhance understanding of patient-specific anatomy in urologic surgery [11], and patient-specific models of the renal vasculature and collecting system have been used both for preoperative planning and as the substrate for simulation and navigation systems [12,13]. By providing a realistic 3D visualisation of the tumour and adjacent structures, 3D models can support preoperative planning for PN. Studies report that surgeons can obtain more detailed information with less effort from 3D models than from standard CT scans [14]. In selected series, reviewing a 3D model has led surgeons to modify their clamping or resection strategy towards a more selective approach, and augmented-reality overlay of such models during robot-assisted PN for complex tumours has been associated with lower rates of global ischaemia and collecting-system violation [15,16].

Despite these benefits, routine use of 3D modelling is limited by practical barriers. High-fidelity reconstructions require high-resolution imaging, specialised software, and technical expertise, often necessitating outsourcing to radiologists or engineers, which can be time-consuming and costly. In an international survey of 100 urologists, 77% reported using 3D models in fewer than 25% of their major operations, identifying cost and the additional time required to obtain the reconstruction as the principal barriers [17]. A range of platforms is now available to support kidney surgery planning [18]. Among them, Synapse3D™ (Fujifilm) offers a user-friendly, clinician-oriented interface with semi-automated segmentation, potentially enabling insourced 3D model generation.

Surgeon-led or in-house reconstruction has been advocated previously, and surveys have documented that urologists perceive 3D models as useful for planning complex renal tumours and would welcome wider availability of the technology [19,20]. What remains largely undocumented, however, are the operational questions that determines whether such a workflow can actually be adopted: how long a clinician without prior experience takes to produce a usable model, how quickly that time falls with experience, and whether the learning trajectory is reproducible across different operators rather than reflecting the aptitude of a single enthusiast. To our knowledge, no previous study has quantified this learning process with more than one operator reconstructing an identical case series.

The present study therefore evaluates the technical feasibility and the learning curve of an insourced, clinician-performed 3D reconstruction workflow using Synapse3D™ in a robotic surgery centre, with each of three previously inexperienced operators reconstructing the same consecutive series. The study was deliberately restricted to the reconstruction process itself and does not address the clinical impact of the resulting models.

## 2. Materials and Methods

### 2.1. Study Design and Patient Selection

This is a single-centre prospective case series that took place at an academic tertiary institution referral for robotic urologic surgery. The study aims to evaluate the use of Synapse3D™ software for insourced 3D imaging reconstruction. To this purpose, consecutive patients scheduled for robotic kidney surgery (both partial and radical nephrectomy) were considered for inclusion within a 30-day time frame (from February to March 2025). The choice of procedure was determined based on tumour size, tumour location, and individual patient risk factors. All procedures were performed using the da Vinci Xi robotic platform (Intuitive Surgical Inc., Sunnyvale, CA, USA), available at our institution. The eligibility criterion for inclusion in the study was the presence of at least one contrast-enhanced CT scan of the abdomen (both arterial and venous phase) with adequate imaging quality and thin slices (≤3 mm thickness).

The study was designed as an exploratory, single-arm feasibility study. In keeping with the conceptual framework for feasibility research [21], no formal sample-size calculation was performed: the sample was defined a priori by a fixed 30-day enrolment window, during which every consecutive eligible patient was included.

The study was approved by the Institutional Ethics Committee (approval ID 7520). All patients signed an informed consent for the collection and use of clinical details and imaging.

### 2.2. Intervention and Workflow

The intervention consisted of an in-house 3D reconstruction of images using the Synapse3D™ software (NAXIS Workstation V6.8.0017EU Build 0052, Fujifilm Corp., Tokyo, Japan) installed on a high-performance imaging workstation in our department.

#### Participants and Training Phase

Three urology residents (postgraduate year 2–4) served as dedicated operators for 3D model reconstruction and participated in the study on a voluntary basis. None had prior hands-on experience with Synapse3D™ or similar 3D reconstruction software before the study. Participants underwent a structured two-day training course provided by Fujifilm application specialists, covering the fundamentals of loading DICOM imaging data, using the Kidney Analysis module for semi-automated segmentation, manual editing of segmentations (for tumour, renal parenchyma, vessels, etc.), and adjustment of visualisation settings to produce a comprehensive 3D model of the kidney. During this training phase, each resident completed eight supervised practice reconstructions on the same set of sample clinical cases, none of which were included in the study. Competency to proceed to the study phase was defined as the ability to complete a full reconstruction, from DICOM import to final rendering, without supervisor intervention.

During the study phase, each of the three residents independently reconstructed all 20 study cases, in the same chronological order, yielding 60 reconstructions in total. Operators worked independently of one another. This fully crossed design was adopted so that the learning trajectory could be assessed within each operator on an identical set of cases, rather than being inferred from cases distributed between operators.

**Figure 1:**
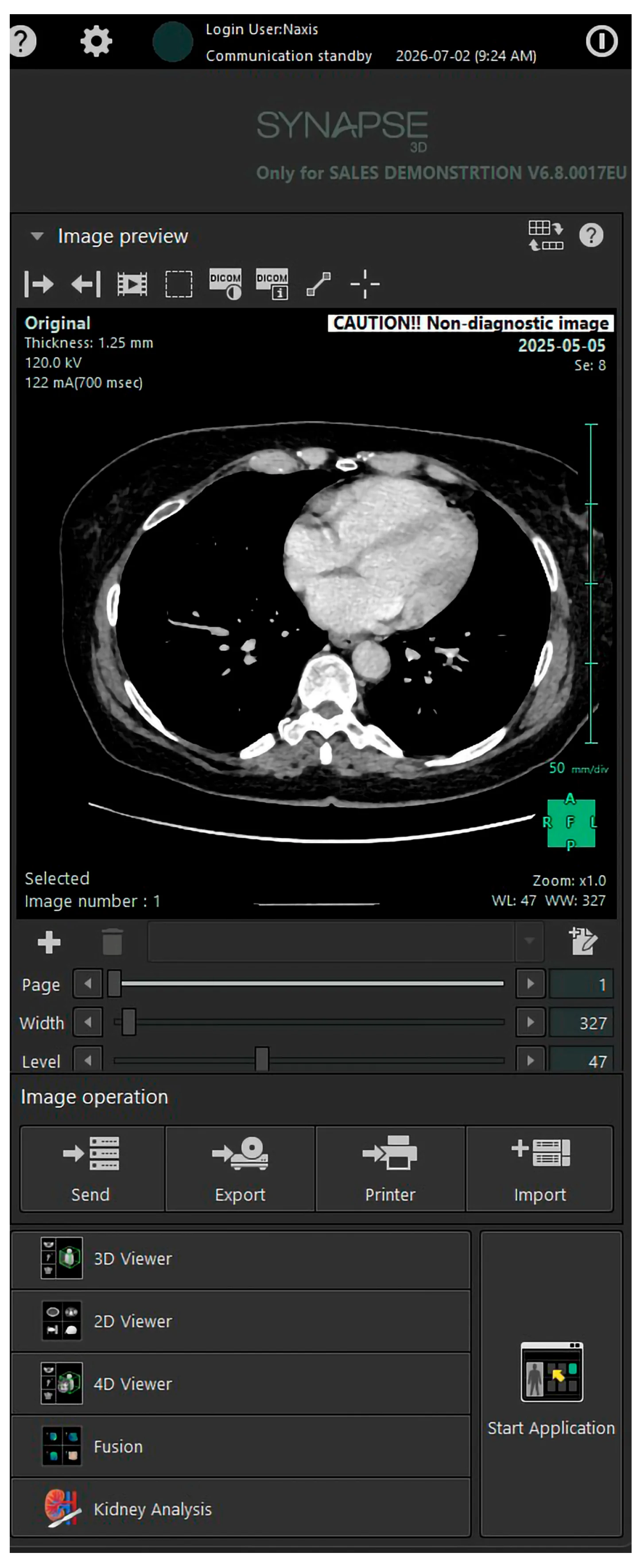
Data import.

**Figure 2:**
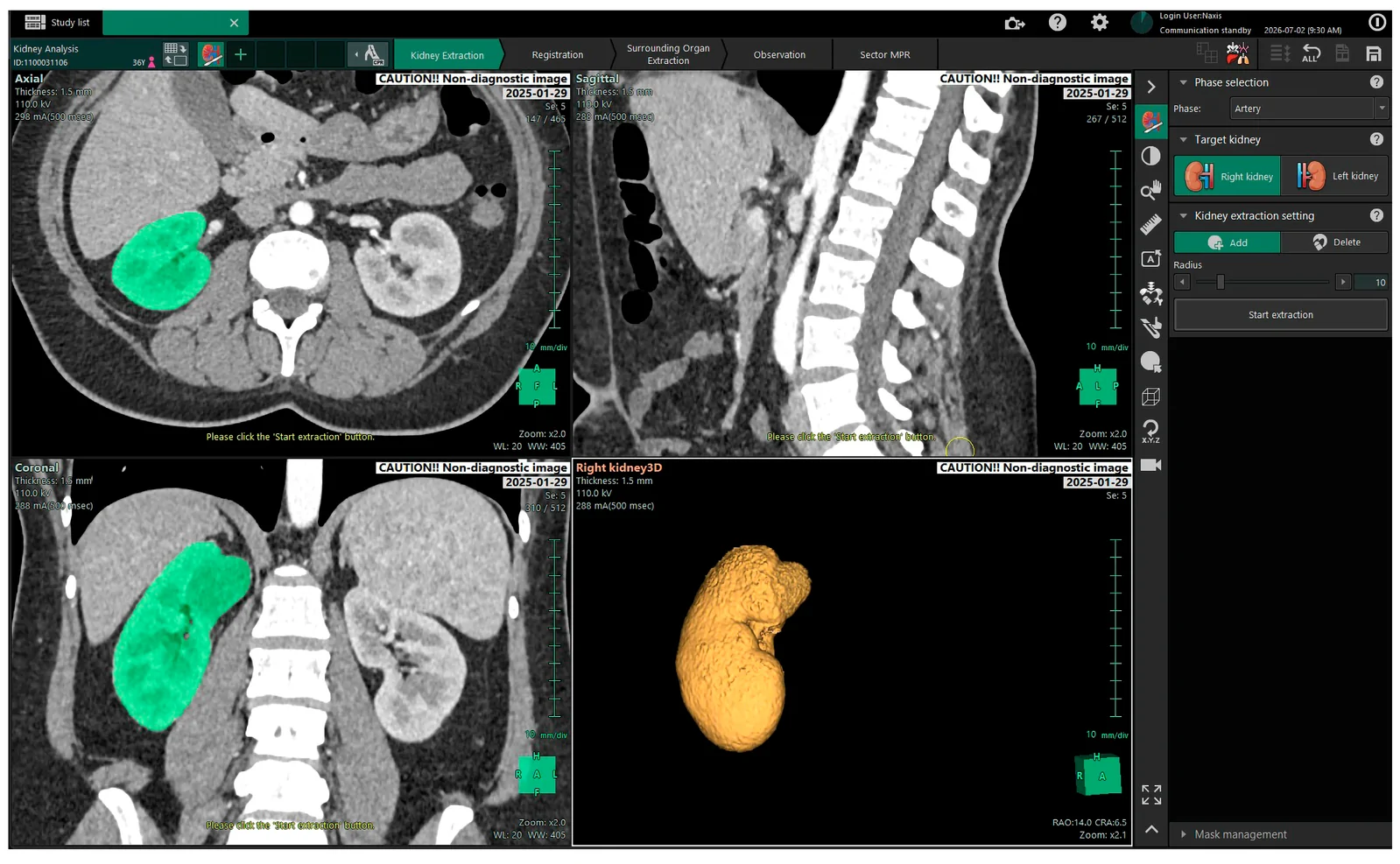
Kidney segmentation (automatic function).

**Figure 3:**
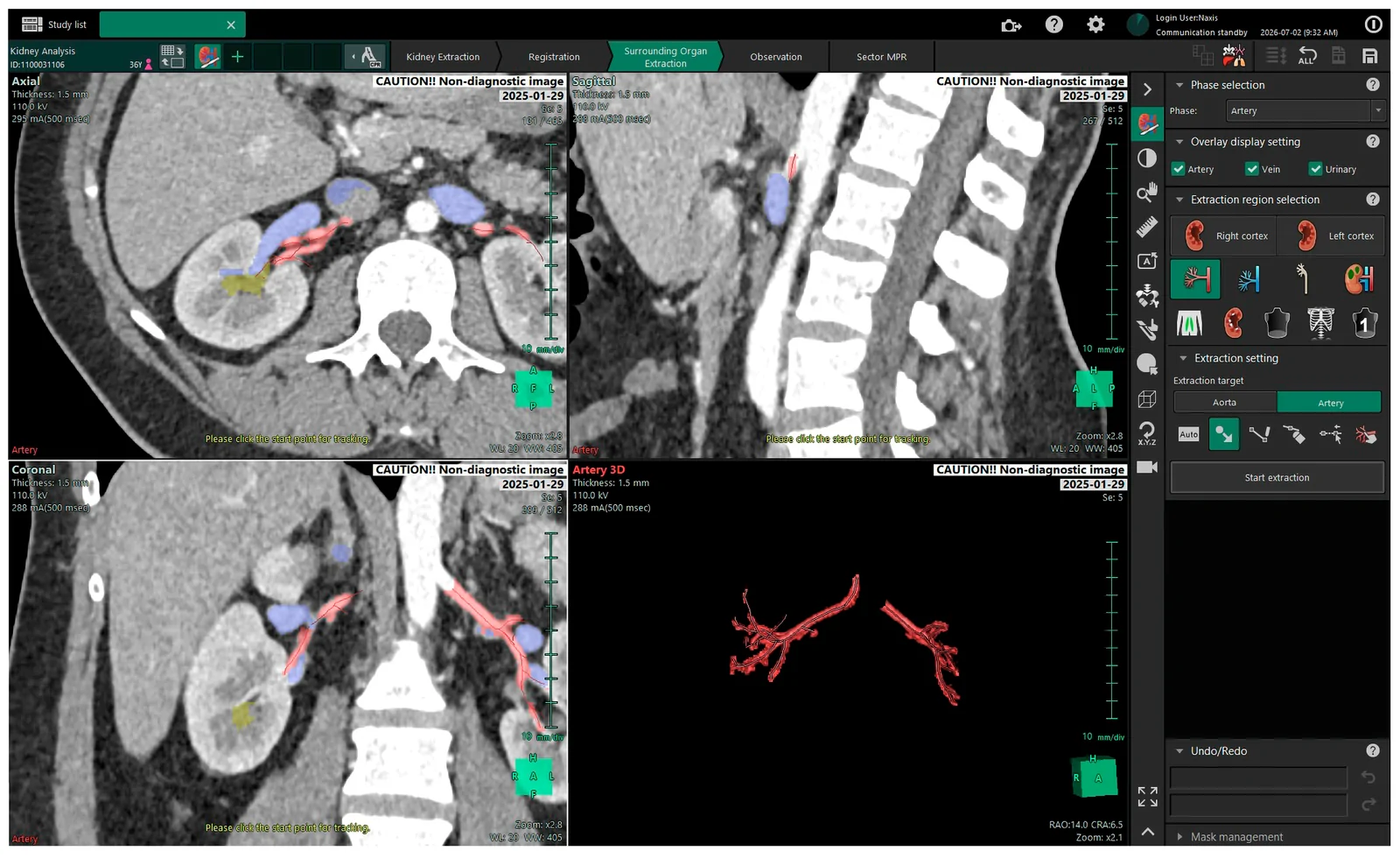
Artery and vein segmentation (semi-automatic function, implementable by the operator).

**Figure 4:**
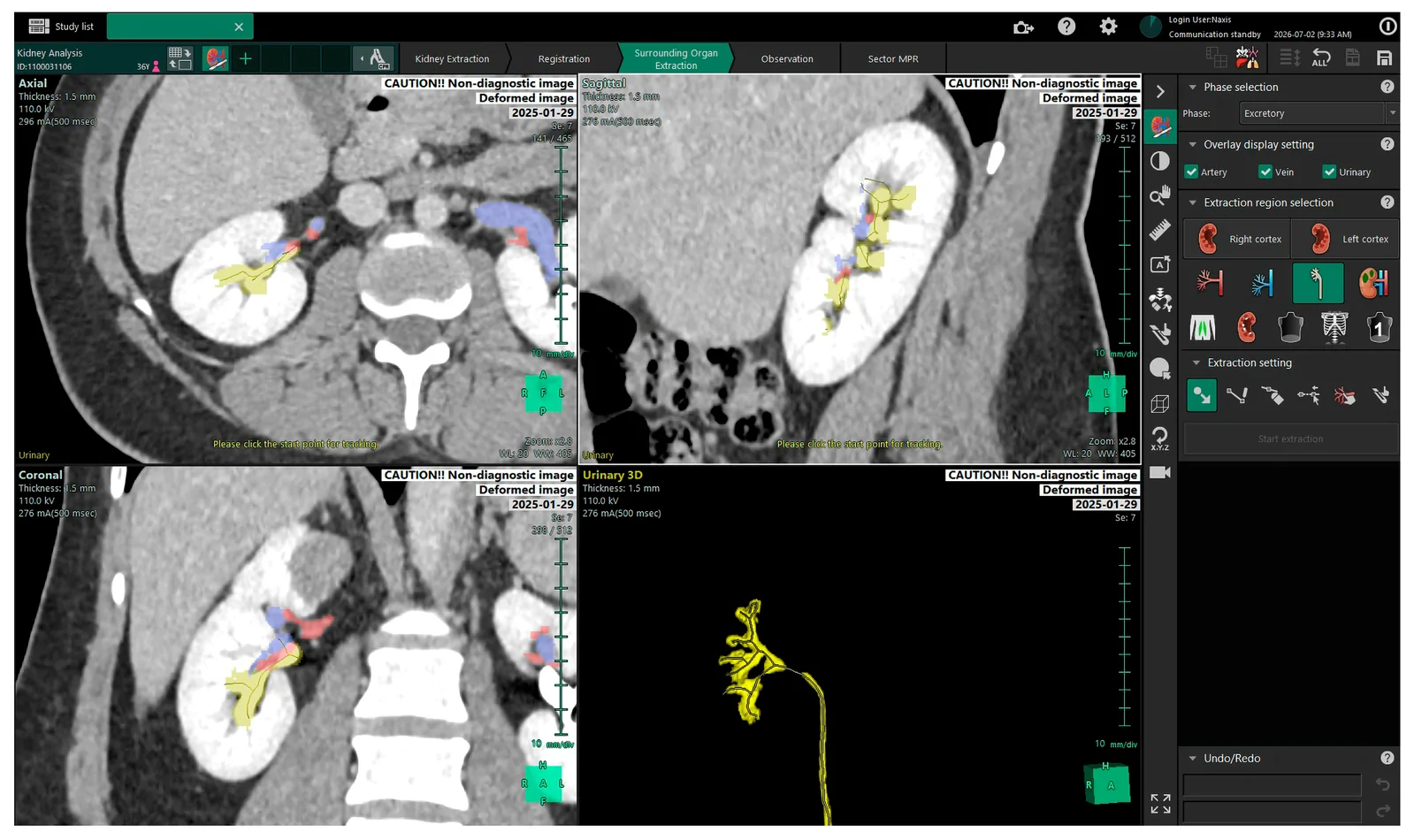
Collecting system segmentation.

For each new patient case, the process was as follows: the anonymised CT DICOM dataset was imported into Synapse3D™ (Figure 1), and the kidney segmentation workflow was initiated (Figure 2). The software typically auto-detects the kidneys and allows the user to delineate the renal cortex, tumour and surrounding organs in successive steps. Key arterial and venous structures were segmented (Figure 3), often starting with an automated algorithm that the user could refine (for example, adding or removing vessel branches). The collecting system (ureteropelvic system) was also segmented (Figure 4). The operator had the ability to toggle between axial, coronal, sagittal views and the developing 3D model to ensure anatomical accuracy. During the reconstruction, the time taken for each case was recorded—measured from the start of the image loading to the final rendering of the 3D model. A stopwatch was used for consistency.

### 2.3. Endpoints

The primary endpoint was the technical feasibility of the insourced workflow, assessed as a composite of the proportion of reconstructions completed successfully, the time required to complete each reconstruction and the need for assistance from the software manufacturer or for repeat segmentation.

**Figure 5:**
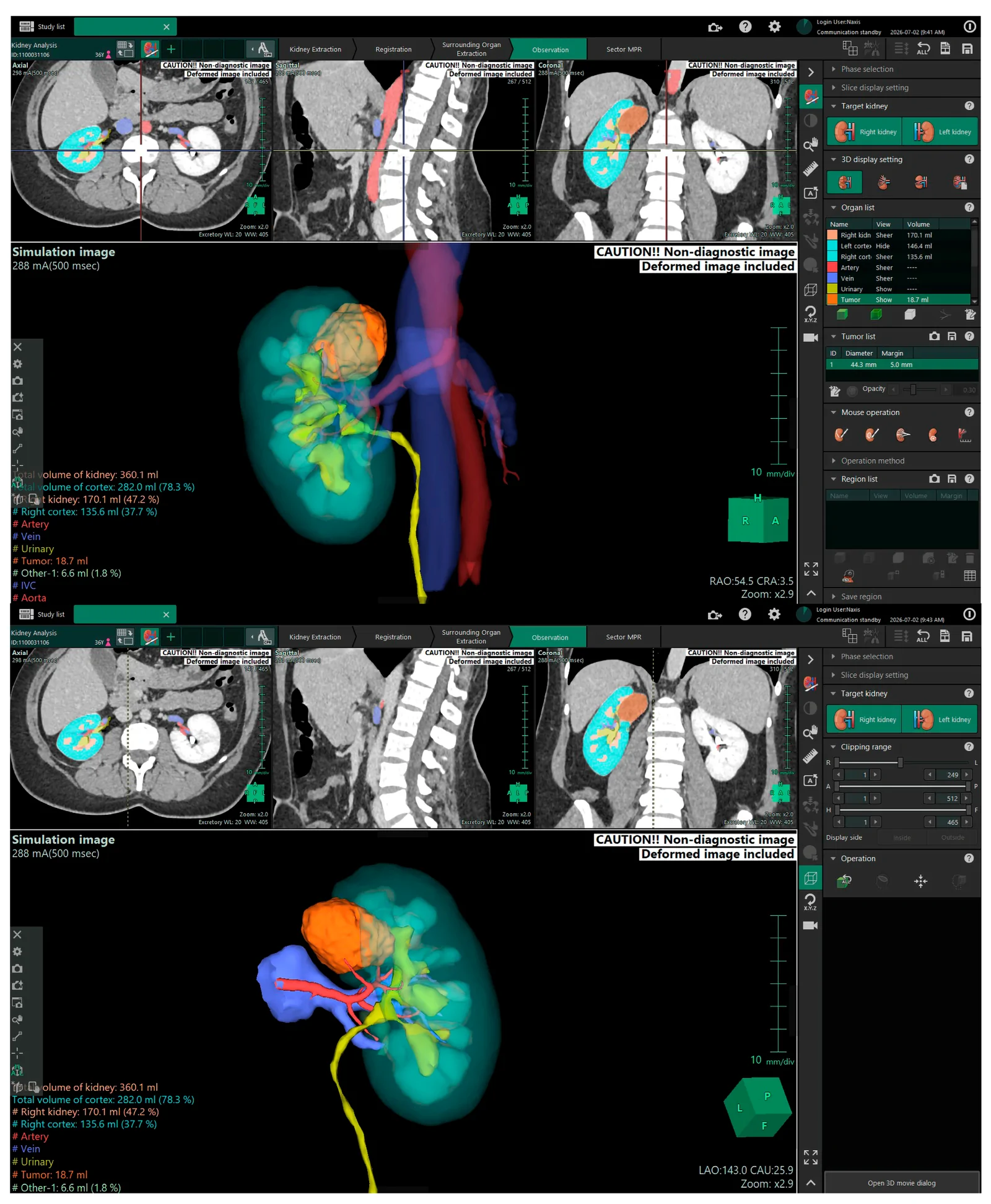
3D model navigator.

As a secondary endpoint, the two experienced robotic surgeons who had performed the operations independently reviewed each 3D model (Figure 5). Neither had been involved in generating the models. The review was deliberately performed after surgery, so that each surgeon could compare the reconstruction against the anatomy encountered in the operative field. Each surgeon rated, on a 5-point Likert scale, the overall image quality, defined as the correspondence between the reconstruction and the intraoperative anatomical findings (1 = very poor, 5 = excellent).

In addition, at the end of the series, each surgeon provided a single global rating of the user-friendliness of navigating the models within the Synapse3D™ interactive viewer, which allowed rotation, zooming and hiding or revealing of individual structures (1 = very difficult, 5 = very easy).

Importantly, the 3D models were not used to define or to modify the surgical strategy in any case: operative planning was carried out conventionally on the basis of cross-sectional imaging, and the reconstructions did not alter decisions already taken. The present study therefore does not evaluate the impact of the models on surgical decision-making.

### 2.4. Statistics

All data were entered in a dedicated database including patient covariates (demographics, tumour size, location and RENAL nephrometry score) and reconstruction covariates (time required to complete each model, in minutes; model assessment scores). Analyses were performed with STATA version 19.0 (StataCorp, College Station, TX, USA).

Continuous variables are reported as median and interquartile range (IQR) and, where useful for comparability with previously published series, also as mean and standard deviation (SD). The predefined comparison between the first and the last ten cases was performed with the Mann–Whitney U test, reporting exact *p*-values. Two-sided *p* < 0.05 was considered statistically significant. Given the sample size, the exploratory design and the number of comparisons performed, all *p*-values should be interpreted descriptively.

## 3. Results

### 3.1. Patient and Tumour Characteristics

Twenty patients were included in the study: 15 males and 5 females, median age 61 years (range 45–72). Seventeen patients underwent robot-assisted PN and three had robotic radical nephrectomy. Median tumour size was 3.0 cm (range 2.5–7.8) with a RENAL (Radius, Exophytic/endophytic, Nearness, Anterior/posterior, and Location) nephrometry score up to 10. Eleven lesions were right-sided and nine left-sided. All CT studies (1–3 mm slice thickness) were successfully imported for reconstruction; no imaging was deemed inadequate. Baseline characteristics are summarised in Table 1, and case-level data are reported in Table 2.

### 3.2. Feasibility of In-House 3D Reconstruction

All 60 reconstructions (three operators × 20 cases) were completed, and each yielded a usable 3D model. No DICOM import errors and no software crashes occurred, no reconstruction had to be repeated, and assistance from Fujifilm application specialists was never required at any point of the study phase. Manual refinement of the semi-automated segmentation was necessary in all cases. Median reconstruction time across the 60 reconstructions was 18 min (range 11–29).

### 3.3. Learning Curve

**Figure 6:**
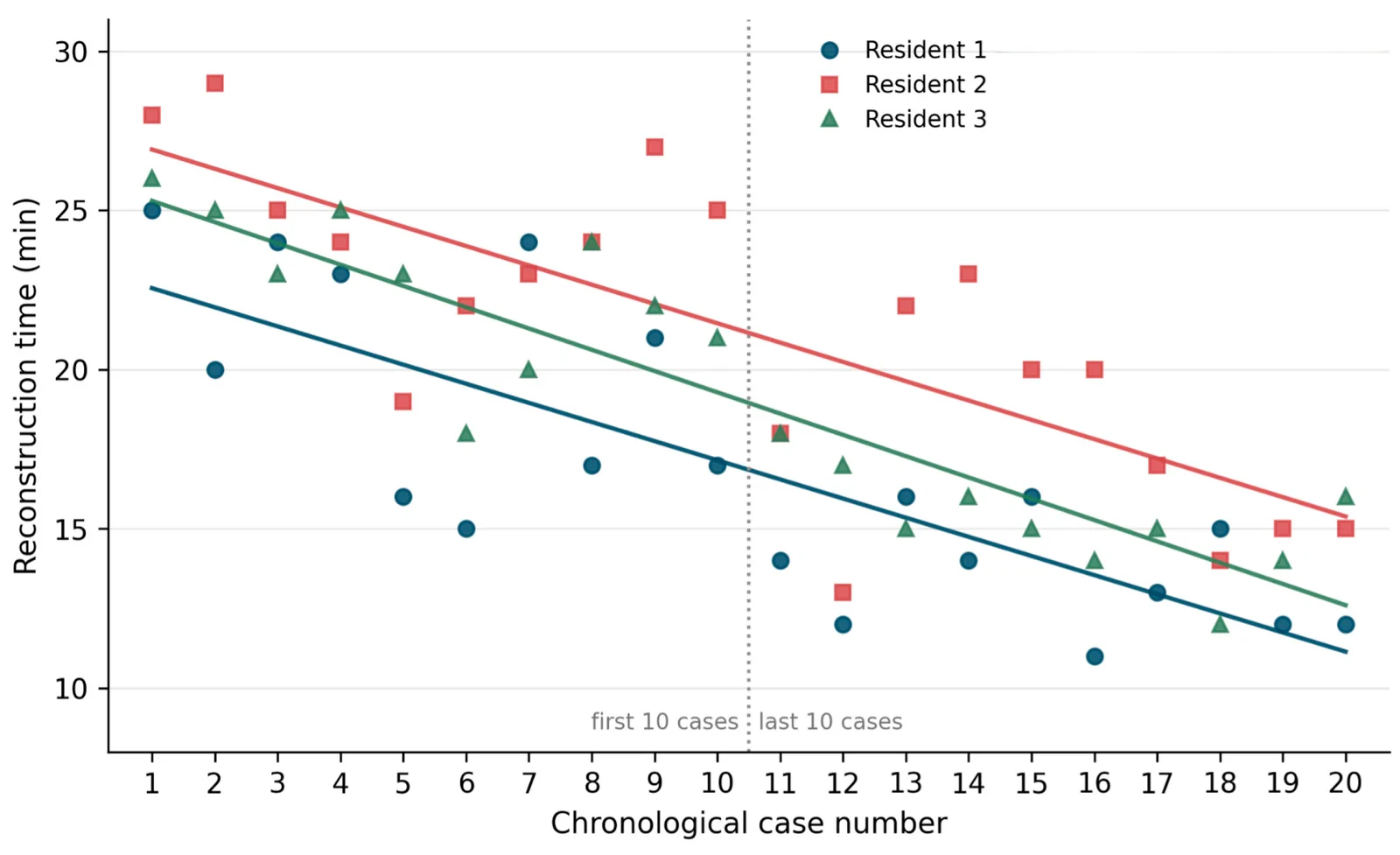
Reconstruction time plotted against chronological case number for each of the three operators. The dotted line separates the first ten from the last ten cases.

Reconstruction time decreased progressively over the series for all three operators (Figure 6). In the regression model adjusted for operator, reconstruction time fell by 0.63 min for each additional case (95% CI 0.51–0.74; *p* < 0.001). Median reconstruction time decreased from 23 min in the first ten cases to 15 min in the last ten (*p* < 0.001). This reduction was significant for each operator analysed separately (Operator 1: median 20.5 to 13.5 min, *p* = 0.0001; Operator 2: 24.5 to 17.5 min, *p* = 0.0003; Operator 3: 23.0 to 15.0 min, *p* < 0.0001), confirming that the effect is not an artefact of pooling data across operators. After approximately ten cases, the mean reconstruction time per case had fallen below 18 min and remained below this value for the remainder of the series.

### 3.4. Model Assessment

Both surgeons rated anatomical fidelity with a median score of 4 (IQR 1). No rating below 3 was recorded. The two surgeons agreed exactly in 7 of 20 cases (35%) and agreed within one point in all 20 cases (100%).

Ratings did not change over the course of the series and were not associated with the RENAL score or with tumour size, indicating that the progressive reduction in reconstruction time was not obtained at the expense of perceived model fidelity.

At the end of the series, the two surgeons rated the overall user-friendliness of navigating the models 3/5 and 5/5, respectively. Both reported that the models depicted tumour–vessel relationships consistently with the intraoperative findings and displayed anatomical variants such as accessory arteries.

## 4. Discussion

The present study shows that an insourced 3D imaging reconstruction workflow is technically feasible and can be acquired rapidly. Every one of the 60 reconstructions was completed successfully, without software failures and without recourse to the manufacturer, and reconstruction time fell by approximately 0.6 min for each additional case. The models were judged to correspond well with the intraoperative anatomy, with a median rating of 4 out of 5 and no rating below 3. The assessment was made after surgery by the operating surgeons themselves. It nonetheless remains a subjective, unblinded, recall-based judgement. Formal validation of insourced models will require quantitative concordance metrics against an independently delineated reference segmentation, or structured intraoperative verification of individual anatomical structures.

A relevant secondary observation is that the reduction in reconstruction time was not achieved at the cost of model quality: ratings showed no downward trend across the series. Speed and fidelity therefore appear to improve, or at least not to trade off against each other, over the first twenty cases.

The insourced approach addresses barriers that have repeatedly been identified as limiting the diffusion of 3D modelling. A workflow that produces a model in roughly a quarter of an hour, on a workstation already present in the department, removes the turnaround component of that barrier. However, no economic data of any kind were collected in this study: no cost comparison with outsourced reconstruction was performed, and the present results therefore support a claim of technical and organisational feasibility, not of cost-effectiveness.

Insourcing also allows the operator’s knowledge of the specific case to be incorporated during segmentation, and the modest time investment can plausibly be absorbed by trainees or dedicated staff. Whether this yields models that are more clinically useful than externally produced ones, and whether the segmentation task itself has educational value for residents, are hypotheses that the present study was not meant to assess.

Synapse3D™ has been used in other contexts that illustrate the analytical potential of insourced segmentation: Leslie et al. [22] derived the renal tumour contact surface area, which correlated with operative time and complication rates, and Motoyama et al. [23] performed volumetric analysis of perinephric fat, reporting an association with console time during robot-assisted PN. Both applications presuppose that segmentation can be performed locally and routinely—the capability examined here.

Compatibility between the Synapse3D™ output and the TilePro™ multi-input display of the da Vinci Xi console was verified in a single case. The model could be displayed alongside the live surgical field, but it could not be manipulated from the console and required an assistant at the workstation to rotate or adjust it. This ergonomic constraint is worth reporting because it determines whether the function is usable in practice. Whether displaying 3D models at the console improves orientation or outcomes was not assessed here and remains to be established; more advanced approaches, in which models are automatically co-registered with the endoscopic image using computer vision or convolutional neural networks, are already under investigation [24] and would make this question more tractable.

### 4.1. Limitations

We acknowledged the following study limitations: First, this is a small, single-centre, single-arm series of 20 patients; the design is exploratory and no sample-size calculation was performed, so the analyses are hypothesis-generating rather than confirmatory. No economic data were collected, so no conclusion regarding superiority or cost-effectiveness can be drawn. Feasibility was assessed descriptively; for confirmatory work, we would propose a composite criterion defined in advance—for example, a completion rate above 95%, a median reconstruction time below 20 min after the tenth case, and no requirement for manufacturer assistance. Finally, the assessment of anatomical fidelity was subjective and unblinded, was performed by the operating surgeons from recall of the operative field.

### 4.2. Future Directions

Future work should address the questions this study was not designed to answer. A formal comparison between insourced and outsourced reconstruction, including turnaround time and a complete cost analysis, is needed before any economic claim can be made. Quantitative validation of anatomical fidelity would replace the subjective endpoint used here. Multicentre replication with a larger number of operators, would establish whether the learning trajectory observed here is generalisable.

## 5. Conclusions

Our preliminary experience demonstrates that insourced 3D image reconstruction using Synapse3D™ is technically feasible: three urology residents without prior experience completed all 60 reconstructions of a consecutive series without failures or manufacturer support. The resulting models were judged by the operating surgeons to correspond well with the intraoperative anatomy. These findings support the technical and organisational viability of generating 3D renal models within a surgical department. They do not, however, establish that the approach is cost-effective, that it improves surgical planning or intraoperative orientation, or that it affects operative outcomes.

## Figures and Tables

**Table 1:** Baseline characteristics of the study cohort.

Characteristic	Value
Age, years (range)	61 (45–72)
Sex—male/female, n	15/5
Side—right/left, n	11/9
Tumour size, cm (range)	3.0 (2.5–7.8)
RENAL nephrometry score—median (IQR)	6 (5–10)
Robot-assisted partial nephrectomy, n (%)	17 (85)
Robot-assisted radical nephrectomy, n (%)	3 (15)
Histopathological diagnosis, n	ccRCC, 20

**Table 2:** Case-level data (Op, operator; RAPN, robot-assisted partial nephrectomy; RARN, robot-assisted radical nephrectomy; S1/S2, assessing surgeon 1 and 2. Cases are listed in chronological order of reconstruction).

Case	Sex	Age	Side	Procedure	Size (cm)	RENAL Score	Op 1 (min)	Op 2 (min)	Op 3 (min)	Fidelity S1	Fidelity S2
**1**	F	45	Right	RAPN	3.8	6	25	28	26	5	4
**2**	F	68	Right	RAPN	7.8	8	20	29	25	4	5
**3**	M	72	Right	RARN	6.6	10	24	25	23	4	4
**4**	M	61	Left	RAPN	2.6	8	23	24	25	5	4
**5**	M	63	Right	RARN	5.0	10	16	19	23	4	5
**6**	M	61	Left	RAPN	2.5	6	15	22	18	5	4
**7**	M	60	Right	RAPN	3.0	6	24	23	20	3	4
**8**	M	64	Right	RAPN	3.4	5	17	24	24	4	3
**9**	F	48	Right	RAPN	3.8	5	21	27	22	4	4
**10**	M	66	Left	RAPN	2.5	5	17	25	21	5	5
**11**	M	45	Right	RAPN	2.8	6	14	18	18	4	4
**12**	F	72	Right	RAPN	4.1	6	12	13	17	4	5
**13**	M	54	Right	RAPN	3.0	5	16	22	15	4	4
**14**	M	50	Right	RAPN	3.6	6	14	23	16	3	3
**15**	F	61	Left	RAPN	2.7	8	16	20	15	5	4
**16**	M	56	Left	RAPN	2.5	6	11	20	14	4	5
**17**	M	68	Left	RAPN	2.6	6	13	17	15	3	3
**18**	M	45	Left	RAPN	3.0	8	15	14	12	5	4
**19**	M	56	Left	RARN	5.5	6	12	15	14	4	5
**20**	M	72	Left	RAPN	2.9	6	12	15	16	3	4

## Data Availability

The raw data supporting the conclusions of this article will be made available by the authors on request.
